# Granzyme A as biomarker for diagnosis in tuberculous pleural effusion

**DOI:** 10.1172/jci.insight.185307

**Published:** 2024-12-06

**Authors:** Fuxiang Li, Chuanzhi Zhu, Yue Zhang, Fanhui Kong, Ximeng Zhang, Liping Pan, Hongyan Jia, Liang Fu, Yunlong Hu, Guofang Deng, Qianting Yang, Xinchun Chen, Yi Cai

**Affiliations:** 1 Guangdong Provincial Key Laboratory of Infection Immunity and Inflammation, Department of Pathogen Biology, Shenzhen University Medical School, Shenzhen, China.; 2 Department of Biochemistry, Center for Molecular Biomedicine, Friedrich Schiller University Jena, Jena, Germany.; 3 Laboratory of Molecular Biology, Beijing Key Laboratory for Drug Resistance Tuberculosis Research, Beijing Chest Hospital, Capital Medical University, Beijing Tuberculosis and Thoracic Tumor Research Institute, Beijing, China.; 4 School of Pharmaceutical Sciences, Shenzhen University Medical School, Shenzhen, China.; 5 Department of Microbiology, Wu Lien-Teh Institute, Harbin Medical University, Harbin, China.; 6 Guangdong Key Lab for Diagnosis & Treatment of Emerging Infectious Diseases, Shenzhen Third People’s Hospital, Shenzhen, China.; 7 National Clinical Research Center for Infectious Disease, Shenzhen Third People’s Hospital, Shenzhen, China.

**Keywords:** Infectious disease, Tuberculosis

## Abstract

**BACKGROUND:**

Current diagnostic tools for tuberculous pleural effusion (TPE) are often inadequate, making accurate diagnosis challenging. Effective identification of TPE is critical for ensuring proper treatment and preventing tuberculosis relapse. This study explored the potential of granzyme A (GZMA) as a biomarker for TPE.

**METHODS:**

Patients with TPE, malignant pleural effusion (MPE), and parapneumonic pleural effusion (PPE) were recruited into discovery and validation cohorts. The discovery cohort consisted of 200 patients with TPE and 100 patients with MPE, while the validation cohort included 167 patients with TPE, 84 patients with MPE, and 69 patients with PPE.

**RESULTS:**

In the discovery cohort, GZMA levels were significantly elevated in TPE compared with MPE, demonstrating 90% sensitivity and 91% specificity at a cutoff of 102.29 ng/mL for effectively distinguishing between the two conditions. In the validation cohort, GZMA maintained high diagnostic performance, distinguishing TPE from MPE with 87% sensitivity and 87% specificity and from PPE with 87% sensitivity and 84% specificity. Incorporating GZMA, lactate dehydrogenase (LDH), and adenosine deaminase (ADA) into a random forest model further improved diagnostic accuracy. In the discovery cohort, this model achieved 92% sensitivity and 100% specificity, and in the validation cohort, it distinguished TPE from MPE with 87% sensitivity and 94% specificity and from PPE with 87% sensitivity and 91% specificity.

**CONCLUSION:**

Overall, GZMA is a promising biomarker for diagnosing TPE, with improved accuracy when combined with LDH and ADA, providing a robust tool for timely identification and effective management of patients with TPE.

**FUNDING:**

The study was supported by Science and Technology Project of Shenzhen (KCXFZ20211020163545004, KQTD20210811090219022, JCYJ20220818095610021, JSGG20220822095200001, JCYJ20210324094614038), Shenzhen Medical Research Funding (B2302035, A2302004), Provincial Natural Science Foundation of Guangdong (2022A1515220034), and Shenzhen Third People’s Hospital Research Foundation (G2022155).

## Abstract 2

Granzyme A (GZMA) is a promising biomarker for diagnosing tuberculous pleural effusion (TPE), achieving high sensitivity and specificity, especially when combined with LDH and ADA.

## Introduction

Pleural effusions (PEs) present a substantial clinical challenge, arising from over 50 identified causes, including local pleural or pulmonary diseases, organ dysfunction, systemic illnesses, and medication effects (1). The formation of PEs typically involves the accumulation of protein-rich fluid and cells within the pleural cavity. The pathophysiological features of PE differ based on the underlying condition. Tuberculosis (TB), malignancies, and bacterial infections such as empyema and pneumonia are the predominant causes of exudative PE (2, 3). Tuberculous PE (TPE), a type of extrapulmonary TB, manifests in a spectrum from benign, self-resolving effusions to more severe forms that may cause pleural thickening, empyema, and pleural fibrosis (4). These complications can lead to chronic PEs and subsequent impairment of lung function (5). Prompt diagnosis and intervention are essential to mitigate the severe outcomes associated with TPE.

TPE, which typically results from a delayed hypersensitivity reaction to subpleural tuberculous lesions, is commonly encountered in clinical practice. Diagnosing TPE presents greater challenges than pulmonary TB due to the difficulty of differentiating it from other PEs, such as malignant PE (MPE) or parapneumonic PE (PPE) (6). The standard diagnostic approach for TPE includes mycobacterial culture, Ziehl-Neelsen staining, and pleural biopsy, often aided by medical thoracoscopy, computed tomography, or ultrasound (7). However, these methods have notable limitations. The detection rates of *Mycobacterium tuberculosis* in pleural fluid (PF) range from 6% to 36% and from 24% to 53% in other specimens. These relatively low detection rates, coupled with the lengthy culture period required for *M*. *tuberculosis*, hinder timely diagnosis (8, 9). While Ziehl-Neelsen staining is highly specific for TPE, its sensitivity is suboptimal (10). Although pleural biopsy can provide critical histopathological insights and positive culture results, it is invasive and its effectiveness largely depends on the expertise of the clinicians performing and interpreting the procedure (11). Compared with traditional methods such as culture, staining, and biopsy, pleural biomarkers offer a cost-effective, noninvasive, rapid, and objective alternative for diagnosing TPE (8, 9). Some biomarkers have been developed to assist diagnosis TPE, such as adenosine deaminase (ADA), IFN-γ, IL-27, tumor necrosis factor-α, IFN-γ–induced protein 10, and GZMK (12). ADA is the most extensively utilized biomarker, known for its high diagnostic accuracy. IFN-γ and IL-27 also demonstrate promising specificity and sensitivity. However, the reliability of other biomarkers continues to require further empirical validation (13).

Granzyme A (GZMA) belongs to granzymes, a family of homologous serine proteases expressed by cytotoxic T lymphocytes, γδ T cells, NK cells, and NK-T cells (14). GZMA is found in the plasma, serum, synovial fluid, and bronchoalveolar lavage fluid in patients experiencing various viral and bacterial infections or other proinflammatory conditions (14). These elevated levels may indicate spontaneous or inadvertent GZMA release due to increased cytotoxic T lymphocyte/NK cell activity in response to persistent inflammation. Importantly, our previous findings revealed an increase in GZMA-expressing cells in PF from individuals with TPE compared with their peripheral blood (12). This elevation suggests that GZMA may play a significant role in the pathogenesis of TPE, potentially influencing the underlying inflammatory processes. Furthermore, the presence of GZMA-expressing cells underscores its potential as a diagnostic biomarker, enhancing our ability to identify and characterize TPE more effectively. This insight encourages us to further investigate GZMA’s applications in clinical diagnostics. To explore this potential, our study investigates whether GZMA is actively produced within the pleural space in cases of PE and assesses the diagnostic value of measuring GZMA levels in PEs.

## Results

### GZMA as a diagnostic biomarker for TPE.

**Figure 1:**
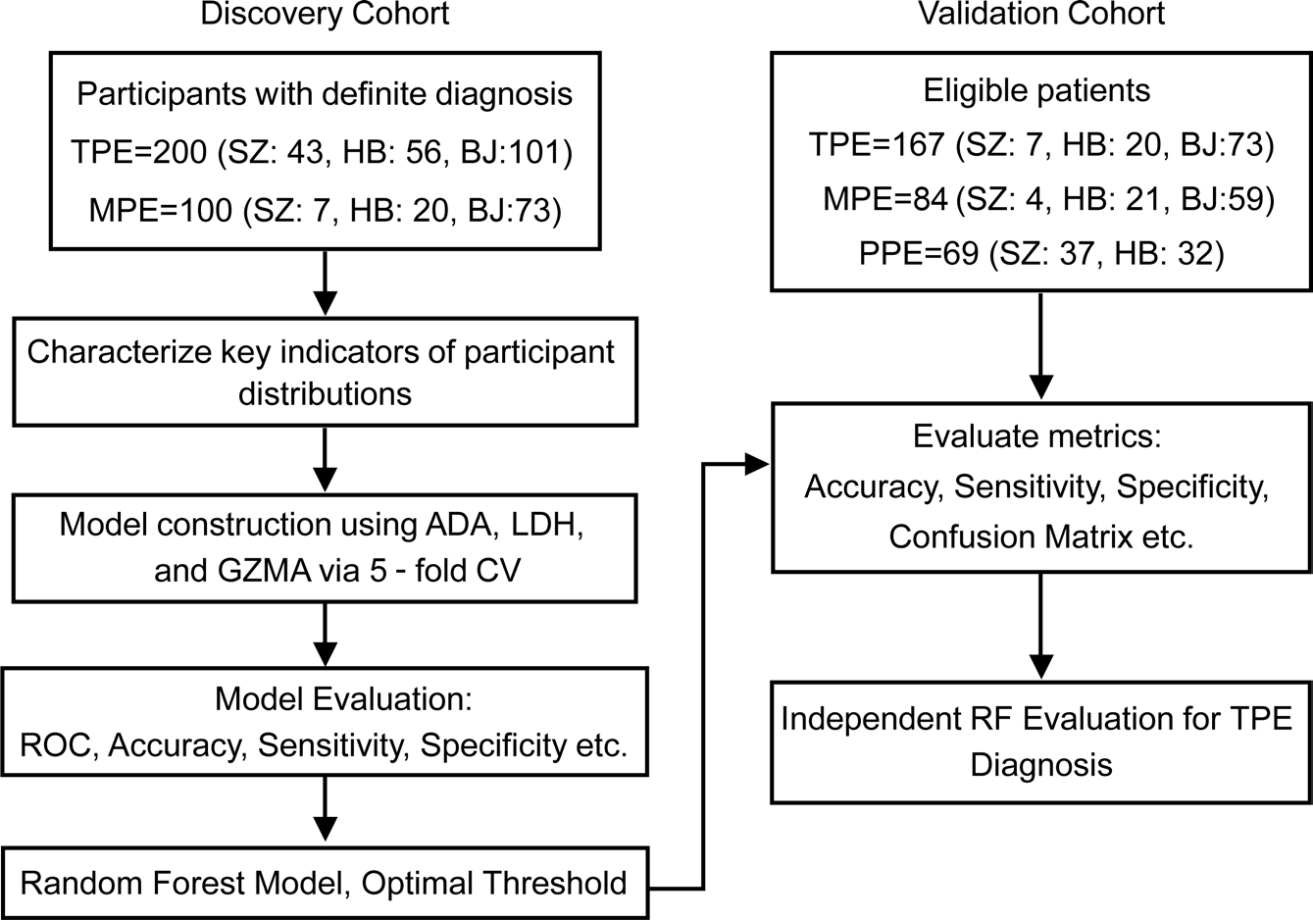
Study design and participant distribution across discovery and validation cohorts. The figure presents a 2-stage study design with discovery and validation cohorts. The discovery phase included 200 patients with TPE and 100 patients with MPE. Among the TPE cases, 43 were from Shenzhen (SZ), 56 were from Harbin (HB), and 101 were from Beijing (BJ). For the MPE cases, 7 were from SZ, 20 were from HB, and 73 were from BJ. Activities included data collection; evaluation of biomarkers, such as ADA, LDH, and GZMA; and development and assessment of predictive models. The random forest model, identified as the most effective, established optimal cutoff values based on ROC curve analysis, accuracy, sensitivity, and specificity. These cutoffs were then tested in the validation cohort, which comprised 167 patients with TPE, 84 patients with MPE, and 69 patients with benign PPE, to evaluate the generalizability and effectiveness of the diagnostic thresholds. Among the validation cohort, TPE included 7 cases from SZ, 20 from HB, and 73 from BJ. MPE included 4 cases from SZ, 21 from HB, and 59 from BJ. PPE included 37 cases from SZ and 32 from HB. Metrics assessed included accuracy, sensitivity, specificity, and confusion matrix analysis.

**Figure 2:**
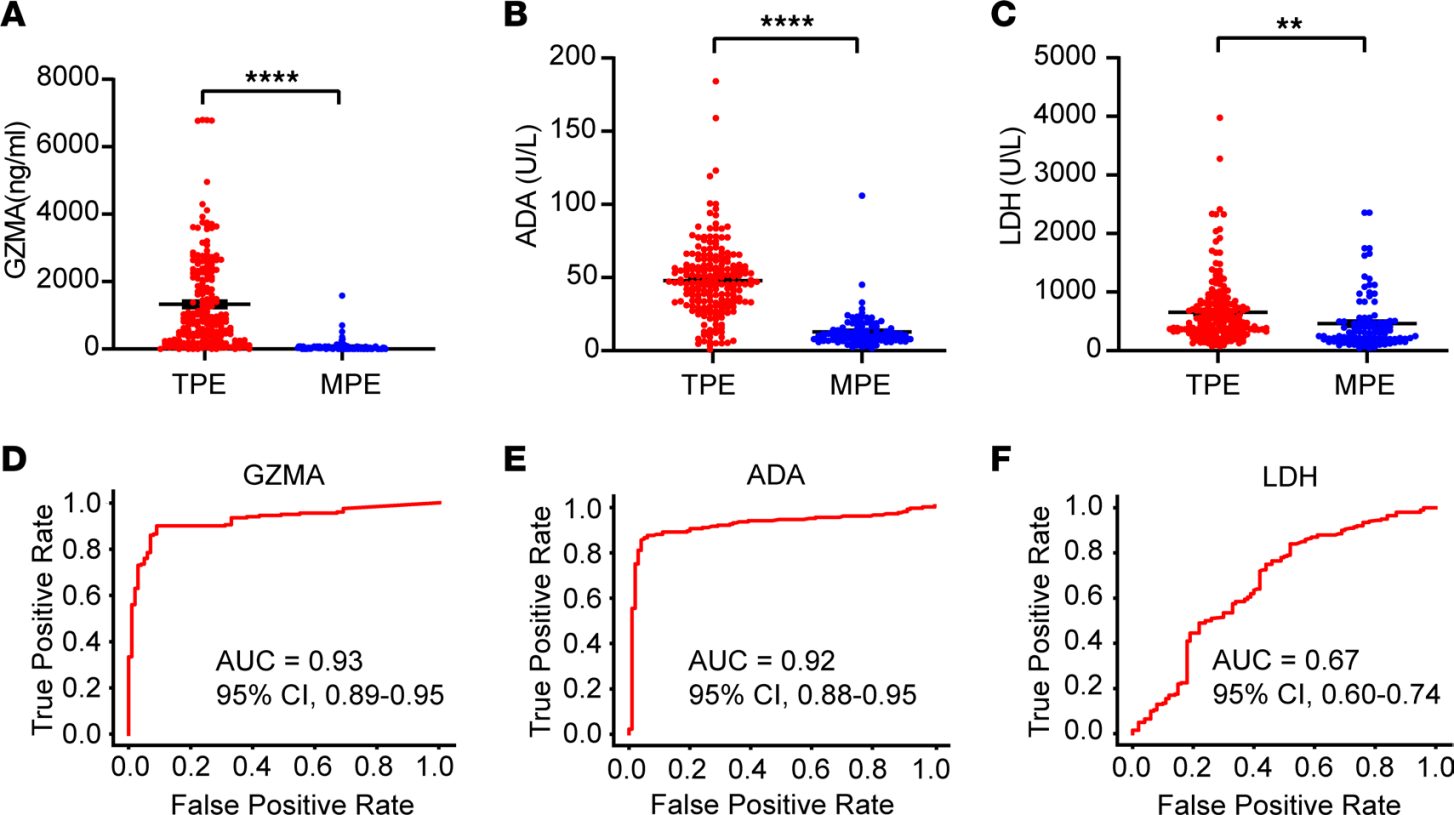
Distributions of ADA, LDH, GZMA, and GZMB in pleural effusion samples and ROC curves for their diagnostic utility in the discovery cohort. (**A**–**C**) The levels of ADA, LDH, and GZMA in pleural effusions from the discovery cohort. Data are shown as the mean ± SEM. Statistical differences between the TPE cohort (*n* = 200) and the MPE cohort (*n* = 100) were analyzed using Student’s 2-tailed unpaired *t* test (**P < 0.01; ****P < 0.0001). *P* values of less than 0.05 were considered significant. (**D**–**F**) The corresponding ROC curves illustrate the trade-off between sensitivity (true-positive rate) and 1-specificity (false-positive rate) for each biomarker, helping to identify optimal cutoff values for diagnosis.

The study involved prospective recruitment from 3 hospitals, dividing participants into 2 cohorts. Clinical and demographic details of the discovery and validation cohort are summarized in Table 1. The discovery cohort included 200 patients diagnosed with TPE and 100 diagnosed with MPE. The cohort used to validate the initial findings comprised 167 patients with TPE, 84 patients with MPE, and 69 patients with PPE (Figure 1). We found that GZMA level was significantly increased in patients with TPE, compared with patients with MPE (Figure 2A), highlighting its potential as a diagnostic marker for TPE. As expected, we observed that the established biomarkers for TPE, ADA, and lactate dehydrogenase (LDH) showed significantly higher levels in patients with TPE compared with patients with MPE in the discovery cohort (Figure 2, B and C).

We therefore further analyzed the diagnosis potential of GZMA in TPE. GZMA demonstrated excellent performance, with a cutoff value of 102.29 ng/mL resulting in an AUC of 0.93 (95% CI, 0.89–0.95), an accuracy of 90% (95% CI, 87%–94%), sensitivity of 90% (95% CI, 86%–94%), and specificity of 91% (95% CI, 85%–97%) (Table 2 and Figure 2D). This establishes GZMA as a valuable biomarker for differentiating between TPE and MPE. Similarly, ADA also demonstrated robust differentiation capabilities with a cutoff of 23.8 U/L, resulting in an AUC of 0.92 (95% CI, 0.88–0.95), an accuracy of 89% (95% CI, 86%–93%), sensitivity of 87% (95% CI, 82%–92%), and specificity of 94% (95% CI, 89%–99%) (Table 2 and Figure 2E). Conversely, LDH displayed limited diagnostic utility, with a median AUC of 0.67 (Figure 2F), underscoring its inadequate accuracy, sensitivity, and specificity (Table 2). In summary, the results revealed high diagnostic accuracy for both GZMA and ADA, while LDH showed lesser performance.

### Enhancing diagnostic accuracy for TPE using a random forest model incorporating GZMA, LDH, and ADA.

To further evaluate the diagnostic accuracy of GZMA alongside clinical ADA and LDH in TPE, several machine-learning models were developed: random forest (RF), logistic regression (LR), gradient boosting machine (GBM), and support vector machine (SVM) (Supplemental Table 1; supplemental material available online with this article; https://doi.org/10.1172/jci.insight.185307DS1). The hyperparameters for these models were optimized using GridSearchCV with 5-fold cross-validation, ensuring data integrity and minimizing overfitting. The models’ performance was validated through bootstrap resampling to establish CIs. Their effectiveness was demonstrated by receiver operating characteristic (ROC) curves, which showed high diagnostic capability with AUC values approaching 1 (15).

**Figure 3:**
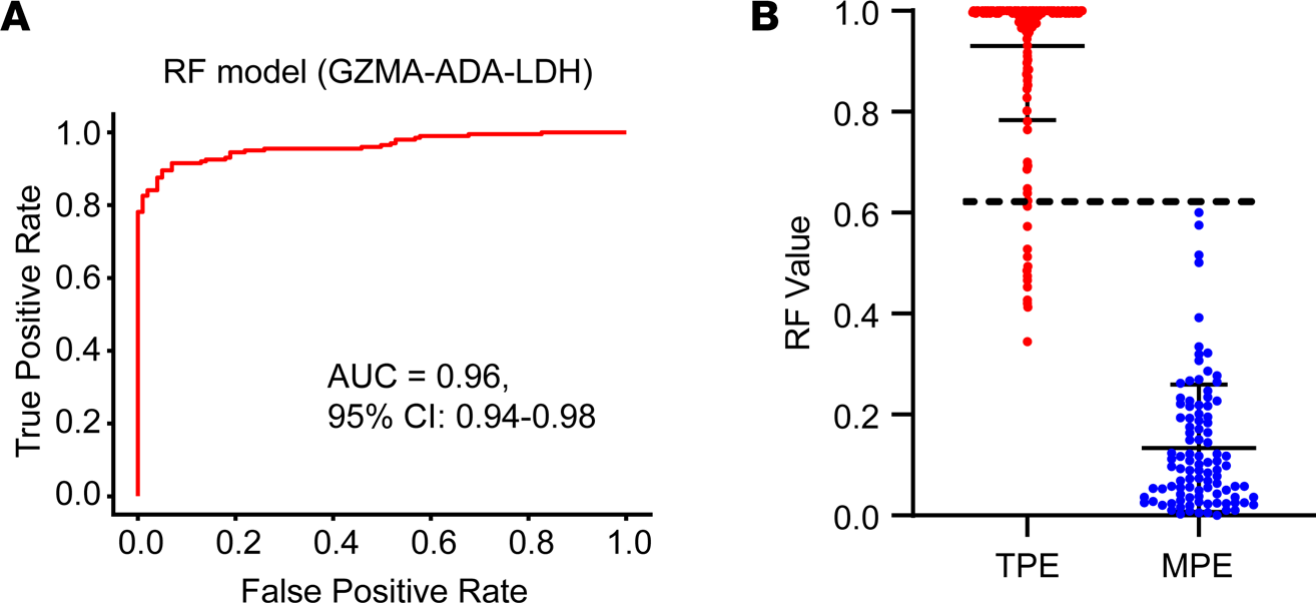
Diagnostic performance of the RF model for TPE using ROC analysis and threshold visualization. (**A**) ROC curves of the RF model utilizing ADA, LDH, and GZMA as features for distinguishing TPE from MPE in the discovery cohort. The AUC for this model is 0.96 (95% CI, 0.94–0.98), indicating excellent diagnostic ability. (**B**) Distribution of the RF model scores for individual samples from both TPE (*n* = 200) and MPE (*n* = 100) groups. The line at *y* = 0.634 represents the optimal cutoff value derived from the RF model, which is used to maximize the sensitivity and specificity for the diagnosis of TPE.

The analysis revealed that the RF model outperformed other predictive models in all key performance metrics (Supplemental Table 1). The RF model implemented a decision threshold of 0.634 for sample classification, effectively distinguishing between TPE (>0.634) and MPE (<0.634) (Table 2 and Supplemental Table 1). At this threshold, the model achieved a median accuracy of 94.7% (95% CI, 92.0%–97.0%), an AUC of 0.96 (95% CI, 0.94–0.98), a sensitivity of 92.0% (95% CI, 88.0%–95.3%), and a specificity of 100% (95% CI, 100%–100%) (Figure 3 and Table 2). While other models also performed well, they did not reach the effectiveness of the RF model. Specifically, the GBR, LR, and SVM models achieved median accuracies of 91.7%, 91.3%, and 90.3%, respectively, and AUC values of 0.94, 0.92, and 0.90, respectively (Supplemental Table 1). Moreover, although using GZMA and ADA independently provided effective differentiation between TPE and MPE, the incorporation of GZMA, ADA, and LDH into the RF model markedly improved its diagnostic accuracy and efficacy (Table 2).

### GZMA and the RF model perform well in an independent validation cohort.

**Figure 4:**
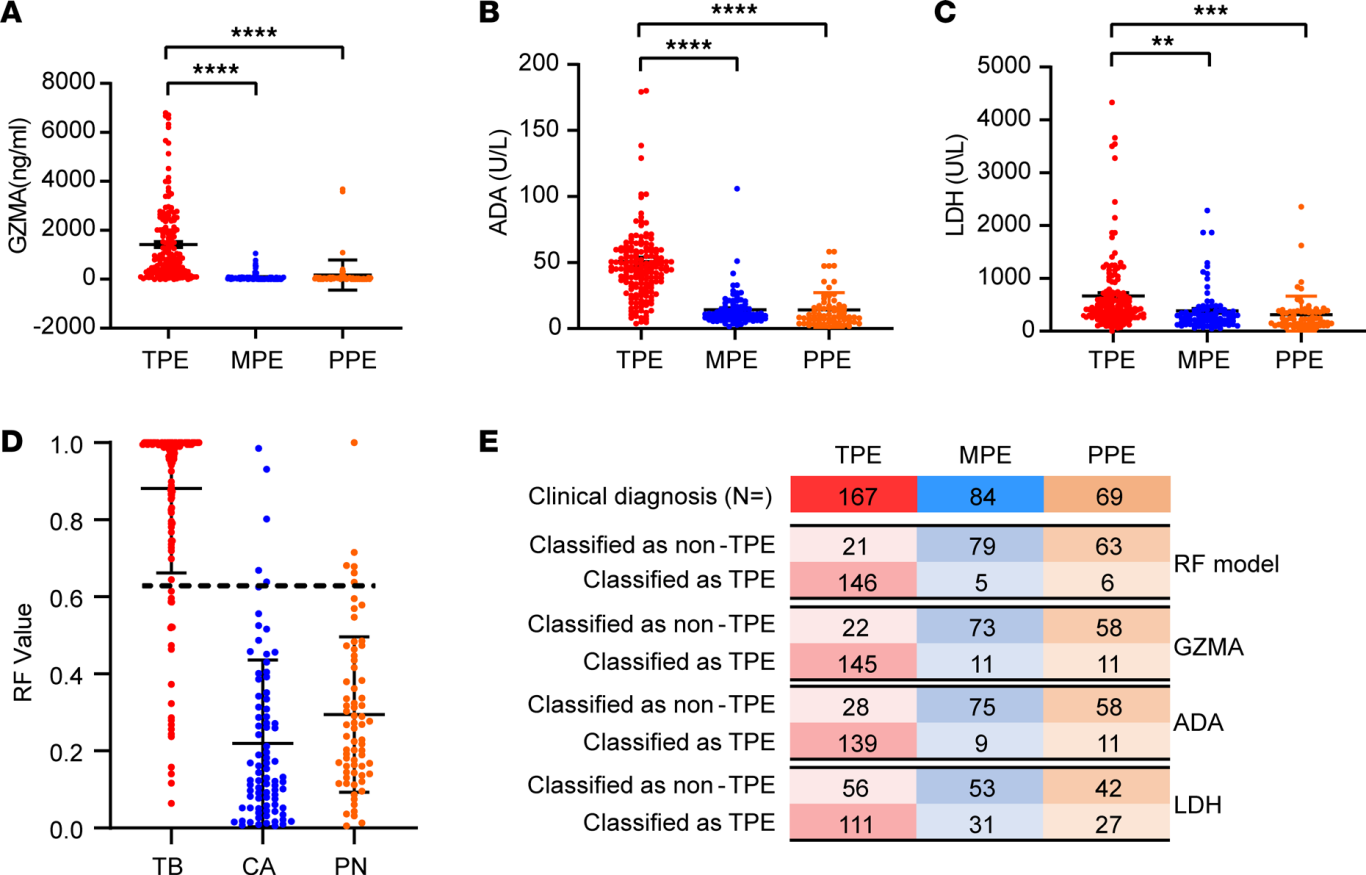
Diagnostic performance of the RF model in an independent validation cohort. (**A**–**C**) The levels of ADA, LDH, and GZMA in pleural effusions from the validation cohort (TPE, *n* = 167; MPE, *n* = 84; PPE, *n* = 69). Data are shown as the mean ± SEM, and differences among the 3 groups were analyzed using 1-way ANOVA with post hoc multiple comparisons (**P < 0.01; ***P < 0.001; ****P < 0.0001). *P* values of less than 0.05 were considered significant. (**D**) A total of 320 participants with clinical diagnosis results were independently validated using the established RF model, with the cutoff value set as 0.634 (indicated by a dotted line). (**E**) Confusion matrices comparing the diagnostic accuracy of ADA, LDH, and GZMA and the RF model for TPE, MPE, and PPE. The RF model shows enhanced performance.

To validate the generalizability of GZMA and the RF model incorporating GZMA, ADA, and LDH for differentiating TPE across independent cohorts, a total of 167 patients with TPE and 84 patients with MPE were recruited for the validation cohort (Figure 1 and Table 1). Additionally, to broaden the diagnostic applicability of GZMA and the RF model, 69 patients with PPE were included in the validation cohort, thereby expanding the evaluation to include a wider range of PEs (Figure 1 and Table 1). In this cohort, the expression levels of GZMA, ADA, and LDH were significantly higher in patients with TPE than in patients with MPE or PPE (Figure 4, A–C).

Applying cutoff values from the discovery cohort, the diagnostic performance of GZMA and ADA was evaluated in the validation cohort. For differentiating TPE from MPE, GZMA demonstrated robust performance with an accuracy of 87% (95% CI, 83%–91%), sensitivity of 87% (95% CI, 82%–92%) and specificity of 87% (95% CI, 80%–94%) (Supplemental Table 2), surpassing ADA in accuracy. In contrast, ADA achieved an accuracy of 85% (95% CI, 81%–90%), sensitivity of 83% (95% CI, 78%–89%), and specificity of 89% (95% CI, 83%–96%) in differentiating TPE from MPE (Supplemental Table 2).

Further analysis employed the same cohort values to assess the ability of these markers to differentiate TPE from PPE. GZMA maintained a high diagnostic accuracy of 86% (95% CI, 82%–90%), with a sensitivity of 87% (95% CI, 82%–92%) and specificity of 84% (95% CI, 75%–93%) (Supplemental Table 3). ADA similarly distinguished TPE from PPE with an accuracy of 83% (95% CI, 79%–88%), sensitivity of 83% (95% CI, 78%–89%), and specificity of 84% (95% CI, 75%–93%) (Supplemental Table 3), which is slightly lower than the accuracy demonstrated by GZMA. These findings affirm the utility of both GZMA and ADA as effective and reliable diagnostic markers for distinguishing TPE from both MPE and PPE.

Although GZMA and ADA individually offer satisfactory diagnostic differentiation among TPE, MPE, and PPE, the RF model, which incorporates GZMA, ADA, and LDH, yields enhanced diagnostic accuracy (Figure 4, D and E; Supplemental Table 2; and Supplemental Table 3). Using a cutoff value of 0.634, the RF model achieves an accuracy of 90% (95% CI, 86%–93%), sensitivity of 87% (95% CI, 82%–92%) and specificity of 94% (95% CI, 89%–99%) for differentiating TPE from MPE (Supplemental Table 2). For distinguishing TPE from PPE, it shows an accuracy of 89% (95% CI, 84%–92%), sensitivity of 87% (95% CI, 82%–92%), and specificity of 91% (95% CI, 84%–97%) (Supplemental Table 3). Thus, while ADA and GZMA effectively differentiate TPE from MPE and PPE using discovery cohort-derived cutoffs, the RF model consistently exceeds the performance of using ADA or GZMA alone.

## Discussion

TPE develops in approximately 30% of patients with TB, leading to substantial impairment in lung function (16). While TPE can sometimes resolve spontaneously, about 65% of these cases advance to active pulmonary TB (7). Therefore, precise and timely diagnosis of TPE is crucial for effective treatment and preventing complications associated with pleural TB. Here, we identified GZMA as a highly effective biomarker for distinguishing TPE from non-TPE cases. When used in combination with ADA and LDH within a RF model, GZMA markedly improved the differentiation of TPE from non-TPEs. This model demonstrated high sensitivity and specificity of 92% and 100%, respectively, which were further validated in an independent cohort. These findings underscore the diagnostic utility of GZMA as a reliable biomarker for TPE. The incorporation of GZMA into diagnostic protocols offers a robust tool for improving the accuracy of TPE diagnosis, facilitating timely and appropriate clinical interventions.

Although ADA is used as a biomarker for diagnosing TPE, its diagnostic utility varies considerably across different geographical regions, clinical settings, and thresholds applied (17). Optimal cutoff values for ADA in diagnosing TPE range widely from 10 to 71 U/L. Increasing the threshold is generally believed to markedly improve ADA’s diagnostic specificity. However, recent meta-analyses have shown that ADA thresholds <36 IU/L, 40 ± 4 IU/L, and 45–65 IU/L yield similar likelihood ratios, suggesting that incremental thresholds do not significantly enhance specificity (17). ADA levels are influenced by various factors. A study found ADA levels >35 U/L in 6% of noncomplicated lung cancer cases, 44% of complicated lung cancer cases, 70% of empyema cases, and 10% of malignant tumors (18). Elevated ADA levels have also been observed in some IgG4 PE cases (19). Age is another critical factor; elderly patients with TPE had lower ADA levels (18.7 ± 10.9 IU/L) (20). Smoking, leukocyte count, CD4^+^ cell count, and certain medications like sunitinib also affect ADA levels (21). Infections and conditions like legionella, brucellosis, and Behcet’s disease can cause high ADA levels (22–24). Conversely, some patients with TPE with confirmed *M*. *tuberculosis* had ADA levels as low as 6.5 IU/L (25). Combining ADA with IFN-γ increases specificity but reduces sensitivity compared with ADA alone (26, 27). While ADA is valuable for diagnosing TPE, it has limitations. In this study, we demonstrated that GZMA effectively differentiated TPE from MPE and PPE with high sensitivity and specificity. When integrated with ADA and LDH, GZMA achieved impressive diagnostic accuracy for TPE versus non-PPE. These results suggest that combining GZMA with ADA and LDH markedly enhances diagnostic precision for TPE.

In the context of TPE, the elevation of GZMA levels may be particularly indicative of the unique immune environment created by *M*. *tuberculosis* or its antigens within the pleural space (5). This environment likely promotes the activation and recruitment of immune cells, leading to the release of granzyme enzymes as part of the immune response to infection (12). This release of GZMA might not only reflect a general inflammatory state but could also be a specific response to the antigenic stimuli presented by *M*. *tuberculosis* within the PE. The exact mechanisms driving the specific elevation of GZMA in TPE remain to be fully elucidated. Further research is needed to dissect the pathways through which GZMA is upregulated in response to *M*. *tuberculosis* antigens and to understand how these mechanisms differ from other conditions (non-TPE) that also show elevated GZMA levels. This research could provide critical insights into the role of GZMA in the immune response to TPE and might identify potential therapeutic targets or diagnostic markers specific to TPE.

GZMA testing demonstrates great potential to become a cornerstone in routine clinical practice for diagnosing TPE (8, 9). Initially, GZMA could function as a rapid and accessible diagnostic tool in primary care, especially in resource-limited countries where advanced diagnostic facilities are lacking. By providing a quick and straightforward method for identifying TPE, GZMA testing can enhance traditional microbiological diagnostics, expediting detection and facilitating faster, more informed decisions regarding invasive procedures. Moreover, GZMA testing is particularly valuable for patients with conditions that naturally elevate ADA levels, offering a robust alternative that ensures accurate and timely diagnosis of TPE, even when ADA results may be ambiguous. However, developing affordable and highly quantitative GZMA assays presents several challenges, including the need for precise measurement techniques, access to central laboratories, and robust quality control. Although GZMA shows promising sensitivity and specificity, its levels can be affected by various clinical factors, pretest probabilities, and generalization, resulting in inconsistencies across different populations and settings. Therefore, further validation in diverse countries and clinical environments is essential to establish the assays’ effectiveness and applicability beyond China.

While GZMA and ADA show strong individual diagnostic performances, integrating these biomarkers into RF models enhances specificity, particularly in complex cases with diagnostic uncertainty or diverse patient populations. In more straightforward cases, individual biomarkers alone may suffice for accurate diagnosis, reducing the necessity for advanced RF models. Despite the RF model’s potential as an automated machine-learning tool for precise diagnostic predictions, its success is highly dependent on the quality of input data, including biomarkers such as ADA, LDH, and GZMA. Regional variability in TB incidence and epidemiological factors can affect pretest probabilities and biomarker levels, creating challenges for consistent model performance. Therefore, further research and validation across various healthcare settings are essential to confirm the model’s broader applicability and accuracy.

In summary, GZMA testing shows promise for improving the diagnostic approach for TPE. Its potential to serve as a rapid, reliable, and accessible diagnostic tool makes it an invaluable asset in both resource-rich and resource-limited healthcare environments. Further research is necessary to validate and enhance the diagnostic capabilities of GZMA and the RF model across larger, more diverse cohorts from various international clinical settings. Such efforts will be crucial to confirm the broad applicability and clinical utility of GZMA testing in diagnosing TPE worldwide.

## Methods

### Sex as a biological variable.

This study involved both male and female patients. Sex was not considered as a biological variable in the analysis. The findings are expected to be applicable to both sexes, as the diagnostic methods and outcomes assessed in this study are not anticipated to differ significantly between male and female individuals.

### Participants.

The patients included in this study had pleural effusions with known causes, such as tuberculosis, malignancy, or parapneumonic effusion; those with pleural effusions of unknown origin were excluded from the study. The 2 study cohorts were recruited from Shenzhen Third People’s Hospital, Harbin Thoracic Hospital, and Beijing Chest Hospital between August 2021 and March 2024. The discover cohort included 200 cases of TPE and 100 cases of MPE. The validation cohort included 167 cases of TPE, 69 cases of PPE, and 84 cases of MPE. The diagnosis of TPE was confirmed if a patient had exudative effusion and was culture positive for *M*. *tuberculosis* (using PF, a pleural biopsy specimen, or sputum) and/or had evidence of TB based on pleural biopsy specimens positive for granulomatous inflammation with acid-fast bacilli. PPE was diagnosed as any effusion associated with bacterial pneumonia, based on the following: (a) lavage fluid or sputum cultures were *M*. *tuberculosis*–negative during clinical follow-up, (b) new infiltration and clinical signs on chest radiography were evident and completely resolved following treatment with appropriate antibiotics, and (c) viral pathogens were not detected. MPEs were diagnosed based on clinical findings and positive cytology in PFs and/or positive histology in the pleural biopsy as well as known malignant disease after exclusion of alternative causes of the PE (28). Patients were excluded if they had received any invasive procedures directed into the pleural cavity or if they had suffered chest trauma within 3 months prior to hospitalization. At the time of sample collection, none of the patients had received any anti-TB therapy, anticancer treatment, antibiotic therapy, corticosteroids, or nonsteroidal antiinflammatory drugs. The characteristics of all cohorts are summarized in Table 1.

### ELISA.

The levels of GZMA in PF samples were determined by ELISA (Multi Sciences, EK1205), according to the manufacturer’s instructions.

### ADA and LDH assay.

The activity of ADA and LDH in PF was measured on a fully automated chemistry analyzer (Beckman Coulter, AU5800). The operation was performed in strict accordance with the standard operating procedure. All experiments were performed in accordance with the manufacturers’ protocols.

### ROC analysis.

ADA, GZMA, and LDH were evaluated as biomarkers to differentiate tuberculous from MPEs. ROC curves for each marker were constructed to assess diagnostic performance. Optimal cutoff values were determined using Youden’s index, with evaluation of diagnostic metrics such as AUC, accuracy, sensitivity, specificity, positive predictive value, and negative predictive value at these thresholds. CIs for these metrics were calculated through bootstrap resampling.

### Model construction and validation.

ADA, GZMA, and LDH were used as biomarkers to construct 4 predictive models: RF, LR, GBM, and SVM. These models were developed, trained, and evaluated within the discovery cohort using the GridSearchCV method. This technique systematically explored combinations of hyperparameters across a stratified 5-fold cross-validation framework, ensuring that each fold represented the class distribution accurately to maintain data integrity. Each iteration involved using 1-fold as the test set (20% of the data), with the remaining 4-folds (80% of the data) used for training. This rigorous validation approach was implemented to ensure robust model evaluations and minimize the risk of overfitting. Bootstrap methods were also employed to determine optimal hyperparameter settings, estimating 95% CIs from 1,000 samples to enhance metric reliability. The best model and its threshold were selected based on comparative analyses of accuracy, AUC, sensitivity, specificity, positive likelihood ratio, negative likelihood ratio, PPV, and NPV. The optimal model and threshold were then applied to the validation cohort to evaluate model efficacy.

### Statistics.

No statistical methods were used to predetermine sample sizes, as direct information on GZMA in this context is unavailable. However, our sample sizes are consistent with those reported in previous publications (29, 30). The exact sample size for each group is provided in the figure legends. Statistical analyses were performed using GraphPad Prism 8. Continuous variables are reported as mean ± SEM and interquartile range (25th–75th percentile). Categorical variables are expressed as frequencies and percentages. The Kolmogorov-Smirnov test assessed the normality of continuous variables. Depending on their distribution, differences between 2 groups were analyzed using either the independent sample Student’s 2-tailed unpaired *t* test or the Mann-Whitney *U* test. For multiple group comparisons, the 1-way ANOVA with post hoc multiple comparisons or Kruskal-Wallis test was applied, contingent on data normality. All hypothesis tests were 2 sided, and a *P* value of less than 0.05 was considered statistically significant.

### Study approval.

This study was approved by the ethics committee of Shenzhen Third People’s Hospital (ethical approval no. 2020-019), the ethics committee of Beijing Chest Hospital, Capital Medical University (ethical approval, no. 2021 and clinical trial review–scientific research, no. 15), and the ethics committee of Shenzhen University Medical School (ethical approval no. PN-202300131). All participants provided written informed consent. All experiments and sampling procedures were conducted in accordance with the ethical and biosafety protocols approved by these institutions.

### Data availability.

Data presented in this manuscript, including values for all data points shown in graphs, are available in the Supporting Data Values file or from the corresponding authors upon request.

## Author contributions

XC, YC, CZ, XZ, and FL conceived and designed the experiments. YC, FL, and XC drafted the manuscript. LF, FL, CZ, YZ, XZ, and LP performed the experiments, and YC, FL, GD, and QY analyzed the data. FK, HJ, and YH provided advice on the project design and data interpretation. The order of co–first authors was determined by the temporal order in which the authors began performing the research. All authors critically reviewed and approved the manuscript.

## Supplementary Material

Supplemental data

ICMJE disclosure forms

Supporting data values

## Figures and Tables

**Table 1:**
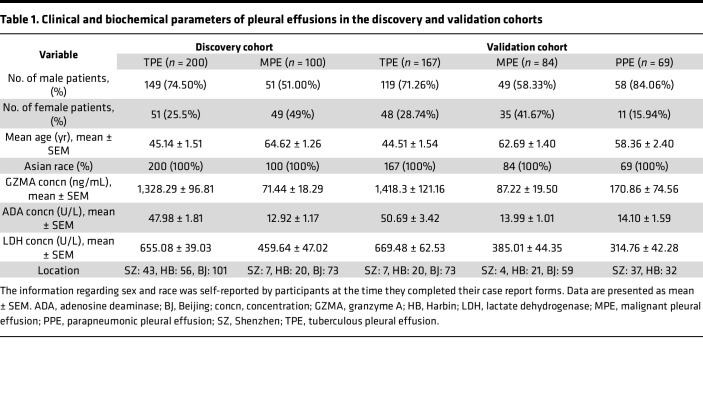
Clinical and biochemical parameters of pleural effusions in the discovery and validation cohorts

**Table 2:**
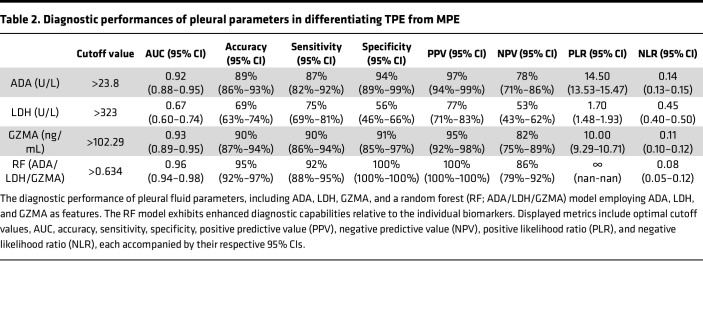
Diagnostic performances of pleural parameters in differentiating TPE from MPE

## Version 1 12/06/2024

Electronic publication
