# Supplementary material for: Granzyme A as biomarker for diagnosis in tuberculous pleural effusion
Source: JCI Insight. 2024 Dec 6;9(23):e185307. doi: 10.1172/jci.insight.185307 (PMC11623936; doi:10.1172/jci.insight.185307)
Supplement: Supplemental data [file jciinsight-9-185307-s239.pdf]

Supplemental Materials for

Granzyme A as biomarker for diagnosis in tuberculous pleural effusion

Fuxiang Li, Chuanzhi Zhu, Yue Zhang, Fanhui Kong, Ximeng Zhang, Liping Pan, Hongyan Jia, Liang Fu, Yunlong Hu, Guofang Deng, Qianting Yang, Xinchun Chen, Yi Cai

This file includes Table S1 to S3.

Sup Table 1: Diagnostic efficiency of different models in diagnosing TPE in the discovery cohort

Model	Cutoff value	Median Accuracy (95% CI)	Median AUC (95% CI)	Median sensitivity (95% CI)	Median specificity (95% CI)	Median PLR (95% CI)	Median NLR (95% CI)	Median PPV (95% CI)	Median NPV (95% CI)
GBM model	0.84	0.92 (0.88-0.95)	0.94 (0.91-0.96)	0.88 (0.83-0.92)	1.00 (1.00-1.00)	∞ (nan-nan)	0.13 (0.08-0.17)	1.00 (1.00-1.00)	0.80 (0.73-0.87)
LR model	0.51	0.91 (0.88-0.94)	0.92 (0.89-0.95)	0.91 (0.86-0.94)	0.93 (0.88-0.98)	12.93 (7.28-36.16)	0.10 (0.06-0.15)	0.96 (0.94-0.99)	0.83 (0.76-0.90)
RF model	0.63	0.95 (0.92-0.97)	0.96 (0.94-0.98)	0.92 (0.88-0.95)	1.00 (1.00-1.00)	∞ (nan-nan)	0.08 (0.05-0.12)	1.00 (1.00-1.00)	0.86 (0.80-0.92)
SVM model	0.48	0.90 (0.87-0.93)	0.90 (0.86-0.94)	0.91 (0.87-0.95)	0.89 (0.83-0.95)	8.27 (5.06-16.41)	0.10 (0.06-0.15)	0.94 (0.91-0.97)	0.83 (0.75-0.90)

The table summarizes diagnostic performance metrics for various predictive models: Gradient Boosting Machine (GBM), Logistic Regression (LR), Random Forest (RF), and Support Vector Machine (SVM). Metrics are calculated at optimal cutoff values and include median accuracy, Area Under the Curve (AUC), sensitivity, specificity, Positive Likelihood Ratio (PLR), Negative Likelihood Ratio (NLR), Positive Predictive Value (PPV), and Negative Predictive Value (NPV). Each metric is presented alongside its 95% confidence interval.

Sup Table 2 Diagnostic capabilities of various indicators for TPE and MPE in the validation cohort.

Variable	Accuracy (95% CI)	Sensitivity (95% CI)	Specificity (95% CI)	PPV (95% CI)	NPV (95% CI)
LDH	0.65 (0.59-0.71)	0.66 (0.59-0.74)	0.63 (0.53-0.73)	0.78 (0.71-0.85)	0.49 (0.39-0.58)
ADA	0.85 (0.81-0.90)	0.83 (0.78-0.89)	0.89 (0.83-0.96)	0.94 (0.90-0.98)	0.73 (0.64-0.81)
GZMA	0.87 (0.83-0.91)	0.87 (0.82-0.92)	0.87 (0.80-0.94)	0.93 (0.89-0.97)	0.77 (0.68-0.85)
RF(GZMA/ADA/LDH)	0.90 (0.86-0.93)	0.87 (0.82-0.92)	0.94 (0.89-0.99)	0.97 (0.94-0.99)	0.79 (0.70-0.87)

This table details the diagnostic performance of lactate dehydrogenase (LDH), adenosine deaminase (ADA), granzyme A (GZMA), and a Random Forest (ADA/LDH/GZMA) model within an independent validation cohort for Tuberculous Pleuritis (TPE) and Malignant Pleural Effusion (MPE). Cutoff values from the discovery cohort were used to evaluate the accuracy, sensitivity, specificity, PPV, and NPV for each biomarker, accompanied by 95% confidence intervals. The RF model demonstrates superior diagnostic precision compared to the individual biochemical markers.

Sup Table 3 Diagnostic capabilities of various indicators for TPE and PPE in the validation cohort.

Variable	Accuracy (95% CI)	Sensitivity (95% CI)	Specificity (95% CI)	PPV (95% CI)	NPV (95% CI)
LDH	0.65 (0.59-0.71)	0.66 (0.59-0.74)	0.61 (0.49-0.72)	0.80 (0.74-0.87)	0.43 (0.33-0.53)
ADA	0.83 (0.79-0.88)	0.83 (0.78-0.89)	0.84 (0.75-0.93)	0.93 (0.88-0.97)	0.67 (0.58-0.77)
GZMA	0.86 (0.82-0.90)	0.87 (0.82-0.92)	0.84 (0.75-0.93)	0.93 (0.89-0.97)	0.72 (0.63-0.82)
RF(GZMA/ADA/LDH)	0.89 (0.84-0.92)	0.87 (0.82-0.92)	0.91 (0.84-0.97)	0.96 (0.93-0.99)	0.75 (0.66-0.84)

This table presents the diagnostic performance of LDH, ADA, GZMA, and the RF (ADA/LDH/GZMA) model for TPE and PPE in the validation cohort. The established

cutoff values from the discovery cohort are used to evaluate the metrics of accuracy, sensitivity, specificity, PPV, and NPV for each indicator. Results demonstrate the RF model's superior diagnostic accuracy relative to the individual markers.
